# Association Between Body Mass Index and Uterotonic Use in Postpartum Hemorrhage: A Retrospective Cohort Study

**DOI:** 10.3390/jcm14176283

**Published:** 2025-09-05

**Authors:** CeCe Cheng, Bryce T. Munter, Michaela Y. Lee, Claire D. Sundjaja, Natasha D. Paul, Margaret M. Klausmeyer, Nastassia A. Yammine, Patrick S. Ramsey, John J. Byrne

**Affiliations:** 1 Department of Obstetrics and Gynecology, Division of Maternal-Fetal Medicine, University of Texas Health Science Center at San Antonio, San Antonio, TX 78229, USA; ccheng1@uw.edu (C.C.); ramseyp@uthscsa.edu (P.S.R.); 2 Department of Obstetrics and Gynecology, University of Texas Health Science Center at San Antonio, San Antonio, TX 78229, USA; bryce.t.munter@uth.tmc.edu; 3 Long School of Medicine, University of Texas Health Science Center at San Antonio, San Antonio, TX 78229, USA; leemi@musc.edu (M.Y.L.); c.sundjaja@dhr-rgv.com (C.D.S.); natasha.paul@vumc.org (N.D.P.); margaret.klausmeyer@hcahealth-care.com (M.M.K.); nastassia.yammine@uth.tmc.edu (N.A.Y.)

**Keywords:** obesity, body mass index, postpartum hemorrhage, management of postpartum hemorrhage

## Abstract

**Background/Objectives:** Our primary objective was to determine whether patients with a higher body mass index (BMI) who experienced postpartum hemorrhage (PPH) required ≥2 uterotonics more often than those with lower BMI. **Methods:** We conducted a retrospective cohort study that included all patients who experienced a PPH between 1 August 2020 and 31 July 2022. Extracted data included patient demographics, PPH risk factors, details regarding the labor course and hemorrhage management, and maternal and neonatal outcomes, such as mode of delivery, etiology of hemorrhage, need for nonpharmacological management, neonatal Apgar scores, requirement for phototherapy, neonatal intensive care unit (NICU) admission, and NICU length of stay. All variables were compared between four BMI classes: non-obese and classes I, II, and III obesity. Possible confounding factors were assessed with a logistic regression analysis. **Results:** Of the 6732 deliveries that occurred during the study period, a total of 891 (13.2%) patients had PPH. Differences were noted in the number of uterotonics used, although no direct correlation was found between increasing BMI class and the use of ≥2 uterotonics. Patients with higher BMIs were more likely to require cesarean delivery, have a classical hysterotomy incision, and have a hysterotomy extension, and were less likely to need a blood transfusion (*p* < 0.05 for all). There was no difference in the rate of uterine atony or other etiologies of hemorrhage, and no difference was observed in the non-pharmacologic management of hemorrhage between groups. **Conclusions:** In our study population, BMI alone does not appear to be directly associated with the use of ≥2 uterotonics.

## 1. Introduction

Obesity, defined as body mass index (BMI) ≥ 30 kg/m^2^, is a significant public health concern affecting one-third of the population [1]. Pregnant individuals with obesity face an increased risk of obstetrical and neonatal complications, including, but not limited to, gestational hypertension, preeclampsia, diabetes, premature delivery, miscarriage, increased need for cesarean delivery, fetal anomalies, fetal macrosomia, and stillbirth [2]. Conflicting data exist on if increasing BMI is associated with higher rates of postpartum hemorrhage (PPH).

Postpartum hemorrhage is a major cause of pregnancy-associated morbidity and mortality worldwide [3,4]. On average, PPH affects 2–5% of all pregnant patients but accounts for 11% of all pregnancy-associated mortalities in the United States [3,5]. Postpartum hemorrhage is defined as blood loss ≥ 1000 mL within 24 h after birth regardless of the route of delivery, based on the reVITALize criteria published in 2014 [6]. To accurately assess blood loss during delivery, more hospital systems have implemented quantitative blood loss (QBL) in place of routinely inaccurate blood loss estimations by physicians and obstetric providers [7]. Prior studies evaluating the correlation with obesity and PPH have utilized estimated blood loss and earlier definitions of PPH; therefore, they may not apply to the current standards set by the American College of Obstetricians and Gynecologists (ACOG) [8,9,10,11,12,13]. These prior studies have yielded mixed results, with several studies demonstrating a correlation between obesity and PPH, while others have found no association. It is also unclear whether there are differences in the management of PPH between patients with increasing BMI, and if delivery outcomes differ by maternal BMI.

We sought to achieve a better understanding of uterotonic use in patients across BMI categories. Our primary aim was defined as the requirement for ≥2 uterotonics during management of a PPH, which would be evaluated across BMI classes.

## 2. Materials and Methods

We conducted a retrospective cohort study of all patients who delivered at our Level IV maternal care hospital from 1 August 2020 to 31 July 2022 and experienced a PPH. This timeframe was selected to capture implementation of QBL and the integration of a new electronic medical record within our hospital system. The inclusion criteria were delivery at our institution and QBL of ≥ 1000 mL within the first 24 h after delivery. Patients were excluded if their medical record was incomplete or lacked information on BMI or QBL.

Medical records were reviewed for sociodemographic characteristics, pre-existing medical conditions, PPH management, and related maternal/neonatal morbidity and mortality. The BMI at the time of delivery was selected as the primary variable due to inconsistent data on pre-pregnancy or early-pregnancy BMI. The study population was divided into four groups based on BMI at the time of delivery: Non-obese: BMI < 30 kg/m^2^, Class I obesity: BMI ≥ 30–34.9 kg/m^2^, Class II obesity: BMI ≥ 35–39.9 kg/m^2^, and Class III obesity: BMI ≥ 40 kg/m^2^ [14].

All patients received at least one uterotonic (oxytocin) per our institutional protocol; therefore, use of additional uterotonic agents or antifibrinolytics was recorded. Non-pharmacologic management of PPH was documented, including transfer to the operating room (if undergoing vaginal delivery), placement of Bakri^®^ balloon (Cook Medical LLC, Bloomington, IN, USA) [15], and need for surgical interventions, such as dilation and curettage, uterine artery embolization, B-Lynch suture placement, O’Leary suture placement, or hysterectomy. For our primary objective, we compared demographic characteristics, uterotonic usage, and nonpharmacologic management of PPH between the four BMI classes. For our secondary objective, we compared the following maternal outcomes—mode of delivery, etiology of the hemorrhage (i.e., Atony, hysterotomy extension, retained products of conception, 3rd/4th degree perineal lacerations), need for blood transfusion or need for non-pharmacologic management of PPH and neonatal outcomes—such as neonatal Apgar scores, requirement of phototherapy, NICU admission, and NICU length of stay—between patients of different BMI classes who experienced a PPH.

Study data were collected and managed using REDCap (Research Electronic Data Capture, Nashville, TN, USA) hosted at our institution [16]. Student’s *t* or Kruskal–Wallis tests were used to compare continuous variables and Fisher’s exact test or Chi-squared analysis were used for categorical variables. Categorical factors were summarized using frequencies and percentages, while continuous variables were summarized using means and standard deviations, or median and interquartile range, depending on normality of the data. GraphPad Prism v10.2.1 for Mac (GraphPad Software, Boston, Ma, USA, www.graphpad.com) and R Studio v4.2.2 (The R Foundation, r-project.org) software were used to perform all statistical analyses. *P*-values < 0.05 were considered significant for two-tailed analysis. For the primary outcome, significant sociodemographic variables including BMI, age, race, ethnicity, gestational age at delivery, anticoagulant use in the antepartum period, induction/augmentation of labor, mode of delivery, and QBL were used in a multivariable logistic regression model to identify the set of covariates that best predict our outcome of interest. This study was approved by our Institutional Review Board (protocol number 20220334EX, approved 20 June 2022 and modified on 19 August 2025, to ensure all data extraction and analysis were appropriately covered under the IRB approval time period).

## 3. Results

**Figure 1:**
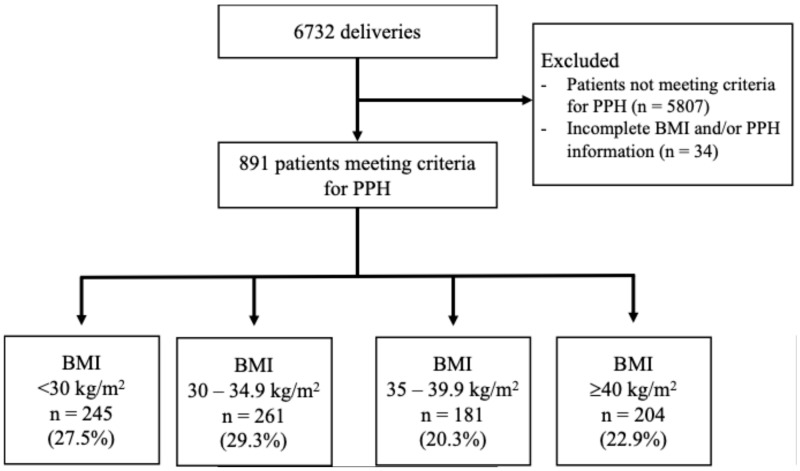
Flow diagram of patient selection for each body mass index class with inclusion and exclusion criteria. BMI, Body mass index; PPH, Postpartum hemorrhage.

Of the 6732 patients who delivered over the study period, 891 patients (13.2%) met criteria for PPH based on QBL ≥ 1000 mL or more within 24 h of delivery and had documented BMI information (Figure 1).

There were significant differences in maternal age, self-identified race & ethnicity, and gestational age at delivery within the study groups (Table 1). Patients with class III obesity were significantly older (*p* = 0.04), less likely to identify as Asian (*p* = 0.003), and more likely to deliver earlier in pregnancy (*p* = 0.003). Additionally, patients with class III obesity had significantly higher baseline rates of pre-gestational diabetes (*p* < 0.0001) and chronic hypertension (*p* < 0.0001) compared patients in the lower BMI groups (*p* < 0.0001). There were no differences in other sociodemographic characteristics (Table 1).

### 3.1. Primary Outcome

Patients with higher BMI were as likely to receive any uterotonic for PPH as those with lower BMI. However, there were significant differences between groups related to the use of ≥2 uterotonics, with the highest rates noted in patients with class I obesity (61.3%), followed by those class II obesity, and those who were non-obese (52.2%, *p* = 0.008, Table 2). Patients with Class III obesity were least likely to receive ≥2 uterotonics. Furthermore, patients with class III obesity received significantly less methylergonovine than the other groups (*p* < 0.0001). There were no differences in administration of misoprostol, carboprost, and tranexamic acid (TXA) between groups.

Multivariable logistic regression analysis showed that patients undergoing IOL (adjusted odds ratio [aOR], 1.63, 95% confidence interval [CI], 1.19, 2.22) and those with a higher QBL (aOR 1.05 per 100 mL increase, 95% CI 1.02, 1.08) were significantly associated with requiring ≥2 uterotonics, while anticoagulant use in pregnancy (aOR 0.26 95% CI 0.09, 0.66) and CD (aOR 0.46, 95% CI 0.34, 0.62) were associated with a significantly lower rate of receiving ≥2 uterotonics (Supplementary Table S1).

### 3.2. Additional Findings

Patients with higher BMIs were significantly more likely to undergo cesarean delivery compared to non-obese patients, with the highest rates observed among those with class III obesity (*p* = 0.0009, Table 3). Additionally, patients with class III obesity were more likely to have a classical hysterotomy (*p* = 0.034) and to have a CD complicated by hysterotomy extension compared to the other groups (*p* < 0.0001). There were no differences in non-pharmacologic management of PPH, such as need to go to the operating room or undergo surgical intervention, between groups. Although QBL at delivery trended higher as obesity class increased, the difference was not significant. There were no differences between groups in total QBL at 24 h. Patients with class III obesity required fewer blood transfusions than those in the lower BMI groups (*p* = 0.002).

There were no significant differences in neonatal birthweight among all groups (Table 4). Neonates born to patients with class III obesity had a higher likelihood of having an Apgar score < 7 at one minute (*p* = 0.009) and five minutes (*p* = 0.02). These neonates also exhibited higher rates of hyperbilirubinemia requiring phototherapy (*p* = 0.003). However, there was no difference between groups in neonatal intensive care unit (NICU) admissions or length of stay in the NICU.

## 4. Discussion

Our data indicate that although differences exist in the requirement of ≥2 uterotonics in the pharmacologic management of PPH, increasing BMI alone is not directly related to the type or number of uterotonics used. There was no difference in surgical or other non-pharmacologic management of hemorrhage within our study population, but patients with a higher BMI showed a decreased need for blood transfusion. There were differences in the type of delivery and cause of postpartum hemorrhage, and in some neonatal outcomes, although not in need for NICU or length of stay.

Contrary to other published studies, the rate of PPH in our study population was not increased in patients with higher BMI, and there was no difference in total QBL. We did not identify a difference in non-pharmacologic management of PPH, which is consistent with the lack of significant difference in QBL among groups. Our results differ considerably from one recent study that demonstrated patients with higher BMI tended to have higher QBL, were more likely to receive an additional uterotonic agent, and were more likely to be moved to the operating room for surgical management of PPH [17]. However, this study used older definitions of PPH and only compared patients with BMI < 30 to those with BMI ≥ 30 kg/m^2^.

After multivariable analysis, neither patient characteristics, such as BMI or age, nor pre-existing conditions, significantly contributed to patients requiring ≥2 uterotonics, which provides additional evidence that obesity itself does not appear to be associated with differences in PPH management. While it seems counterintuitive that anticoagulant use in pregnancy significantly decreased the risk of requiring ≥2 uterotonics, anticoagulant use may affect coagulation status more than uterine atony and therefore is not associated as closely with uterotonic use. Furthermore, patients on anticoagulation may also have other medical comorbidities precluding uterotonic use. Similarly, while CD was previously thought to be associated with higher QBL and more uterotonic use, in our study, it was found to be protective. This difference might be explained by the more rapid recognition of uterine atony during CD, non-atonic etiologies of PPH, and improved ability to perform non-pharmacologic procedures for management of PPH. Furthermore, our data suggests that rates of non-atonic etiologies of PPH, such as hysterotomy extension, are higher in those with higher BMI, and higher BMI is directly correlated with the amount of QBL. However, these etiologies are not treated by uterotonics or even TXA, but by surgical management. Based on our data, intrapartum factors such as IOL or augmentation of labor and mode of delivery contribute more to the management of PPH compared to antepartum characteristics.

Differences in maternal outcomes were noted and consistent with previously published literature, where patients with higher BMI required delivery via cesarean more often. The association between maternal obesity and both classical hysterotomy and hysterotomy extension in surgery has not been noted in literature directly. Some physicians perform vertical midline skin incision over Pfannenstiel incision in patients with morbid obesity, and this vertical skin incision is associated with classical hysterotomy [18,19]. However, this study did not address the type of skin incision. Classical hysterotomy is also associated with delivery at earlier gestations. Hysterotomy extension has been shown in the previous literature to not be related to maternal obesity but is associated with having labored prior to cesarean [20,21]. We did not control for gestational age at the time of delivery or by indication for delivery.

We also found that patients with higher BMIs had a decreased need for blood transfusion. This counterintuitive finding has been seen in certain surgical procedures such as hip and knee arthroplasty, and coronary bypass surgery, but is not universal, and has not yet been demonstrated in pregnant patients undergoing delivery [22,23]. Several factors may contribute to this phenomenon. From a physiological standpoint, non-pregnant patients with obesity have increased lean body weight and adipose tissue, resulting in a higher metabolic demand and potentially improved oxygen delivery [24]. Furthermore, obese patients may have increased blood volume, despite being proportionally lower than overall weight, resulting in a smaller blood volume loss during surgery [25]. It is unclear if the same pattern of lean mass and blood volume increase is seen in pregnant patients with obesity [26]. If this is accurate for pregnant patients, those with obesity may be able to withstand a greater volume of blood loss prior to necessitating transfusions. It is also important to note that higher BMI is associated with a more prothrombotic state, which contributes to a hypercoagulable state and may affect blood loss and need for transfusions [27].

We observed statistically significant differences in Apgar scores and rates of hyperbilirubinemia across the BMI categories; however, these differences did not correspond to increased rates of admission to the neonatal intensive care unit. Our findings were consistent with prior publications on the impact of obesity on Apgar scores and hyperbilirubinemia. One systematic review and meta-analysis demonstrated patients with BMI 30–40 kg/m^2^ had increased risk of Apgar scores < 7 at 1 and 5 min (OR 1.28, OR 1.34, respectively) and those with BMI ≥ 30 kg/m^2^ had even higher risk (OR 1.63, OR 1.66) [28]. Researchers have postulated this may be due to increased placental lipid accumulation in maternal obesity and the expected, associated inflammation and oxidative stress in the placenta [28]. Association between maternal obesity and neonatal hyperbilirubinemia has been less studied, although our data is consistent with one study published from Hawaii demonstrating that maternal obesity was associated with increase in both maternal and neonatal bilirubin levels due to inhibition of enzymes that function in maternal bilirubin metabolism [29].

### Strengths and Limitations

A limitation of our study is its retrospective and single-institution nature. Furthermore, our institution provides care for a primarily Hispanic/LatinX population, thus potentially limiting generalizability to all obstetric populations. Additionally, our data was limited because it included only certain variables that are related to PPH. For example, we did not have information on coagulation factor levels on admission to labor and delivery since it is not our practice to routinely collect these labs upon admission for labor. Finally, it is important to note that we use BMI at the time of delivery rather than pre-pregnancy BMI, which may not accurately reflect the combined effects of weight gain and BMI during pregnancy. Despite these limitations, several strengths of this study are worth noting. Our study included a large patient population and equal distribution of patients with all classes of obesity, whereas previous studies usually only compared patients with BMI < 30 kg/m^2^ to those with BMI ≥ 30 kg/m^2^. Our study was also performed in a Level IV maternal designation center, where a standardized PPH protocol was utilized, QBL was deployed rather than EBL, and the reVITALize criteria and updated definitions for PPH were applied.

## 5. Conclusions

Our data suggest that BMI itself is not directly associated with the need for more uterotonics, which has significant clinical implications. This can inform obstetric care providers on appropriate counseling and management of their pregnant patients with higher BMIs in the peripartum period. Given the rising national rates of obesity, this becomes increasingly important. Future studies may consider evaluating the pharmacokinetics of uterotonics in pregnant people with different BMIs.

## Figures and Tables

**Table 1:** Demographic Characteristics.

	Non-Obese BMI <30 kg/m^2^ (n = 245)	Class I: BMI 30–34.9 kg/m^2^ (n = 261)	Class II: BMI 35–39.9 kg/m^2^ (n = 181)	Class III: BMI ≥40 kg/m^2^ (n = 204)	*p* Value
Age (years)	28 (23, 32.8)	29 (24, 34)	29 (24, 35)	30 (25, 35)	**0.04**
BMI (kg/m^2^)	27.4 (25.4, 29)	32.3 (31.2, 33.6)	37.1 (36.1, 38.6)	44.2 (42.2, 49.2)	**<0.0001**
Self-Identified Race and Ethnicity					
Hispanic/LatinX	171 (69.8)	200 (76.6)	146 (80.7)	160 (78.4)	0.45
Non-Hispanic/LatinX					
- White	34 (13.9)	31 (11.9)	17 (9.4)	17 (8.3)	0.24
- Black	17 (6.9)	11 (4.2)	10 (5.5)	21 (10.3)	0.06
- Asian	17 (6.9)	12 (4.6)	2 (1.1)	3 (1.5)	**0.003**
- Other/unknown	6 (2.4)	7 (2.7)	6 (3.3)	3 (1.5)	0.64
Nulliparous	104 (42.4)	106 (40.6)	82 (45.3)	80 (39.2)	0.64
EGA (weeks)	39.1 (37.4, 40.1)	39 (37.6, 40.1)	39 (37.1, 39.9)	38.6 (37, 39.4)	**0.0003**
Admission Hgb (g/dL)	11.6 (10.4, 12.6)	11.9 (10.8, 12.5)	11.8 (10.7, 12.4)	11.4 (10.6, 12.1)	0.11
Commercial insurance	74 (39.2)	70 (26.8)	49 (27.1)	50 (24.5)	0.6
Tobacco use	8 (3.3)	8 (3.1)	4 (2.2)	9 (4.4)	0.69
Anticoagulant use during pregnancy	4 (1.6)	4 (1.5)	6 (3.3)	10 (4.9)	0.11
Required induction or augmentation of labor	133 (54.3)	144 (55.2)	112 (61.9)	118 (57.8)	0.74
Pre-existing medical condition					
- Pre-gestational DM	2 (0.8)	12 (4.6)	16 (8.8)	23 (11.3)	**<0.0001**
- Asthma	5 (2)	10 (3.8)	8 (4.4)	11 (5.4)	0.3
- **Chronic HTN**	6 (2.4)	18 (6.9)	18 (9.9)	31 (15.2)	**<0.0001**

Kruskal–Wallis test for nonparametric continuous data. Chi-squared and Fisher’s exact test used for categorical data. Values presented as median [P25, P75], n (%). BMI, body mass index; Self-Identified Race, Other, includes American Indian/Hawaiian PI, more than one race, & Unknown; Hgb, hemoglobin; DM, diabetes mellitus; HTN, hypertension. *p* Values: Bold values suggest *p* < 0.05.

**Table 2:** Uterotonic management of Hemorrhage by BMI class.

Pharmacologic Management	Non-Obese: BMI <30 kg/m^2^ (n = 245)	Class I: BMI 30–34.9 kg/m^2^ (n = 261)	Class II: BMI 35–39.9 kg/m^2^ (n = 181)	Class III: BMI ≥40 kg/m^2^ (n = 204)	*p* Value
Any uterotonic used	236 (96.3)	254 (97.3)	176 (97.2)	199 (97.6)	0.87
≥2 uterotonics	128 (52.2)	160 (61.3)	99 (54.7)	93 (45.6)	**0.008**
Misoprostol	50 (20.4)	60 (23)	30 (16.6)	29 (14.2)	0.08
Carboprost	88 (35.9)	95 (36.3)	69 (38.1)	57 (27.9)	0.14
1 dose	56 (22.9)	49 (18.8)	37 (20.4)	30 (14.7)	0.18
2 doses	20 (8.2)	29 (11.1)	21 (11.6)	18 (8.8)	0.55
≥3 doses	12 (4.9)	17 (6.5)	11 (6.1)	9 (4.4)	0.72
Methylergonovine	111 (45.3)	121 (46.4)	61 (33.7)	43 (21.1)	**<0.0001**
Tranexamic acid	130 (53.1)	160 (61.3)	112 (61.9)	110 (53.9)	0.11

Kruskal–Wallis test for nonparametric continuous data. Chi-squared and Fisher’s exact test used for categorical data. Values presented as n (%). BMI, Body mass index. *p* Values: Bold values suggest *p* < 0.05.

**Table 3:** Maternal Outcomes.

	Non-Obese: BMI <30 kg/m^2^ (n = 245)	Class I: BMI 30–34.9 kg/m^2^ (n = 261)	Class II: BMI 35–39.9 kg/m^2^ (n = 181)	Class III: BMI ≥40 kg/m^2^ (n = 204)	*p* Value
Mode of delivery					
- Cesarean Delivery	119 (48.6)	143 (54.8)	107 (59.1)	137 (67.2)	**0.0009**
Uterine incision					
- Low transverse	103 (42)	123 (47.1)	100 (55.2)	108 (52.9)	**0.027**
- Classical	14 (5.7)	12 (4.6)	6 (3.3)	20 (9.8)	**0.034**
- Other	3 (1.2)	9 (3.4)	2 (1.1)	8 (3.9)	0.13
Reason for PPH					
- Atony	107 (43.7)	130 (49.8)	85 (47)	82 (40.2)	0.19
- Hysterotomy extension	26 (10.6)	24 (9.2)	32 (17.7)	45 (22)	**<0.0001**
- Retained POCs	2 (0.8)	3 (1.1)	3 (1.6)	3 (1.5)	0.89
- 3rd or 4th degree perineal laceration	10 (4.1)	7 (2.7)	2 (1.1)	4 (2)	0.52
Nonpharmacologic Management of PPH					
- Vaginal packing	15 (6.1)	10 (3.8)	7 (3.9)	5 (2.5)	0.26
- Bakri	13 (5.3)	22 (8.4)	9 (5)	9 (4.4)	0.24
- UAE	5 (2)	3 (1.1)	0 (0)	2 (1)	0.27
- Move to OR ^1^	7 (5.7)	9 (7.6)	6 (3.3)	3 (4.4)	0.77
- D&C	2 (0.8)	4 (1.5)	2 (1.1)	1 (0.5)	0.77
- B-lynch	2 (0.8)	1 (0.4)	2 (1.1)	3 (1.5)	0.64
- O-Leary	17 (6.9)	6 (2.3)	10 (5.5)	12 (5.9)	0.1
- Hysterectomy	11 (4.5)	5 (1.9)	4 (2.2)	3 (1.5)	0.22
QBL at delivery (mL)	1168 (1000, 1431)	1152 (1003, 1393)	1116 (1006, 1434)	1245 (1031, 1507)	0.053
Total QBL at 24 h (mL)	1312 (1137, 1624)	1310 (1130, 1568)	1272 (1130, 1618)	1384 (1142, 1665)	0.59
Required blood transfusion	86 (35.1)	64 (24.5)	45 (24.9)	40 (19.6)	**0.002**

Kruskal–Wallis test for nonparametric continuous data. Chi-squared and Fisher’s exact test used for categorical data. Values presented as median [P25, P75], n (%). BMI, Body mass index; CD, cesarean delivery; PPH, postpartum hemorrhage; POCs, products of conception; QBL, quantitative blood loss. ^1^ Vaginal delivery only, n = 122, 118, 73, 67 for BMI < 30 kg/m^2^, BMI 30–34.9 kg/m^2^, BMI 35–39.9 kg/m^2^ and BMI ≥ 40 kg/m^2^, respectively. Other uterine incision includes low vertical, T, J, and high transverse hysterotomies *p* Values: Bold values suggest *p* < 0.05.

**Table 4:** Neonatal Outcomes.

	Non-Obese: BMI <30 kg/m^2^ (n = 245)	Class I: BMI 30–34.9 kg/m^2^ (n = 261)	Class II: BMI 35–39.9 kg/m^2^ (n = 181)	Class III: BMI ≥40 kg/m^2^ (n = 204)	*p* Value
Neonatal birthweight	3334 (2865, 3665)	3380 (2950, 3780)	3374 (2983, 3780)	3440 (2933, 3790)	0.18
Apgar Scores					
- 1 min < 7	39 (15.9)	36 (13.8)	25 (13.8)	51 (24.5)	**0.009**
- 5 min < 7	15 (6.1)	9 (3.4)	6 (3.3)	19 (9.3)	**0.02**
NICU admission	51 (20.8)	56 (21.5)	40 (22.1)	51 (25)	0.62
Hyperbilirubinemia requiring phototherapy	44 (18)	39 (14.9)	39 (21.5)	58 (28.4)	**0.003**
Neonate LOS (days)	12.5 (4.75, 34.5)	12 (5, 23)	12.5 (4.75, 32.75)	18 (5, 37)	0.51

Kruskal–Wallis test for nonparametric continuous data. Chi-squared and Fisher’s exact test used for categorical data. Values presented as median [P25, P75], n (%). NICU, neonatal intensive care unit; LOS, length of stay. *p* Values: Bold values suggest *p* < 0.05.

## Data Availability

The data is not publicly available online.

## Supplementary Materials

The following supporting information can be downloaded at: https://www.mdpi.com/article/10.3390/jcm14176283/s1, Table S1: Logistic regression analysis for primary outcome: use of ≥2 uterotonics.
