# Supplementary material for: Association Between Body Mass Index and Uterotonic Use in Postpartum Hemorrhage: A Retrospective Cohort Study
Source: J Clin Med. 2025 Sep 5;14(17):6283. doi: 10.3390/jcm14176283 (PMC12428878; doi:10.3390/jcm14176283)
Supplement: Supplementary file 1 [file jcm-14-06283-s001.zip › jcm-3758129-supplementary.pdf]

Supplementary Table S1. Logistic regression analysis for primary outcome: use of ≥2 uterotonics

Independent variable	Univariate analysis		Multivariable analysis	
	OR (95% CI)[a]	P value	aOR (95% CI)	P value
**Body mass index**	0.98 (0.97, 0.99)	**0.039**	0.99 (0.97, 1.01)	0.4
- <30kg/m^2 (Reference)	-	-		
- 30 – 34.9kg/m^2	1.45 (1.02, 2.06)			
- 35 - 39.9kg/m^2	1.11 (0.76, 1.64)			
- ≥40kg/m^2	0.76 (0.52, 1.10)			
**Age**	0.96 (0.94, 0.98)	**<0.001**	0.98 (0.96, 1.01)	0.14
- ≤24 (Reference)	-	-		
- 25-34	0.73 (0.53, 1.0)			
- 35-44	0.57 (0.39, 0.84)			
- ≥45	0.21 (0.01, 1.70)			
**Hispanic/LatinX Ethnicity**				
- No (Reference)	-	-	-	-
- Yes	1.41 (1.04, 1.92)	**0.029**	1.28 (0.85, 1.94)	0.2
**White Race**				
- No (Reference)[b]	-	-	-	-
- Yes	1.54 (1.06, 1.92)	**0.018**	1.33 (0.82, 2.15)	0.2
**Gestational age**	1.05 (1.02 – 1.09)	**0.005**	1.04 (1.00, 1.09)	0.081
**Admission Hgb (g/dL)**	1 (0.97 – 1.002)	0.25		
**Anticoagulant use in pregnancy**				
- No (Reference)	-	-	-	-
- Yes	0.28 (0.10, 0.67)	**0.004**	0.28 (0.10, 0.71)	**0.011**
**Cesarean delivery**				
- No (Reference)	-	-	-	-
- Yes	0.37 (0.28, 0.49)	**<0.0001**	0.47 (0.35, 0.64)	**<0.001**
**Low transverse hysterotomy**				
- No (Reference)	-	-		
- Yes	0.84 (0.51, 1.39)	0.5		
**HTN**				
- No (Reference)	-	-		
- Yes	0.94 (0.7 - 1.25)	0.66		
**Diabetes**				
- No (Reference)	-	-		
- Yes	0.88 ((0.64, 1.2)	0.41		
**VB on admission**				
- No (Reference)	-	-		
- Yes	1.23 (0.79, 1.94)	0.36		
**IOL/Augmentation**				
- No (Reference)	-	-	-	-
- Yes	2.15 (1.65, 2.84)	**<0.001**	1.57 (1.14, 2.15)	**0.005**
**TOLAC**				
- No (Reference)	-	-		
- Yes	1.01 (0.57, 1.80)	0.98		
**Thrombocytopenia <150**				
- No (Reference)	-	-		
- Yes	1.45 (0.94, 2.27)	0.09		
**EFW or neonatal weight >4000**				
- No (Reference)	-	-		
- Yes	1 (0.69, 1.46)	>0.99		
**QBL**[c]	1.04 (1.01, 1.07)	**0.001**	1.05 (1.02, 1.08)	**0.002**

**Hgb < 10g/dL or Hct <30%**			
-	No (Reference)	-	-
-	Yes	1.05 (0.72, 1.53)	0.8
**Placenta abnormality**			
-	No (Reference)	-	-
-	Yes	0.9 (0.46, 1.8)	0.77

HTN, hypertension; *PPH,* postpartum hemorrhage; *VB,* vaginal bleeding; *EFW,* estimated fetal weight; *PAS,* placenta accreta spectrum; *Hgb,* hemoglobin; *Hct,* hematocrit; *CD,* cesarean delivery; *TOLAC,* trial of labor after cesarean; *IOL,* induction of labor; *IAI,* intra-amniotic infection

[a]OR = Odds Ratio; CI = Confidence interval
[b]Non-white race was selected as the reference given our primarily Hispanic/LatinX population
[c]Per 100ml change

P Values: Bold values suggest p<0.05
